# Targeting the miR-32533/CREB5 axis: a promising immunomodulatory and therapeutic strategy for Alzheimer’s disease

**DOI:** 10.3389/fimmu.2026.1780328

**Published:** 2026-05-04

**Authors:** Lizhen Li, Xiaolin Zhao, Li Zeng, Zixuan Li, Kaiyue Zhao, Zhongdi Cai, Rui Liu

**Affiliations:** Institute of Medicinal Biotechnology, Peking Union Medical College and Chinese Academy of Medical Sciences, Beijing, China

**Keywords:** Alzheimer’s disease, amyloid-beta, miR-32533/CREB5 pathway, neuroinflammation, oxidative stress, therapeutic strategy

## 1 Introduction

Alzheimer’s disease (AD) is the leading cause of dementia worldwide, and its prevalence is projected to triple by 2050, posing a substantial societal and economic burden (1–3). The pathological hallmarks of AD include the accumulation of amyloid-beta (Aβ) plaques, neurofibrillary tangles of hyperphosphorylated tau (p-tau), and a persistent state of neuroinflammation (4–8). While recent Food and Drug Administration (FDA) approvals of anti-Aβ immunotherapies such as Lecanemab and Donanemab represent a pivotal advancement (9), their effects are modest and primarily target a single aspect of the disease cascade. It is well-established that Aβ aggregates elevate intracellular reactive oxygen species (ROS), leading to oxidative damage and establishing a vicious cycle that further reinforces Aβ aggregation and oxidative stress (10). Simultaneously, Aβ activates glial cells, triggering neuroinflammation marked by elevated levels of pro-inflammatory cytokines such as tumor necrosis factor-alpha (TNF-α) and interleukin-6 (IL-6), which in turn exacerbate Aβ dyshomeostasis and synaptic impairment (11–13). The synergistic interplay between oxidative stress and neuroinflammation, perpetuated by Aβ, accelerates disease progression, highlighting the pressing need for therapeutic interventions that can concurrently address multiple pathological pathways, including Aβ dysmetabolism, oxidative stress, and chronic neuroinflammation.

In this context, microRNAs (miRNAs) have emerged as attractive candidates due to their ability to precisely regulate complex gene networks (14–16). The recent identification of a novel miRNA, miR-32533, and its intricate involvement in AD pathogenesis through the cyclic-AMP response binding protein 5 (CREB5) axis presents a fresh avenue for investigation (16). In this opinion article, we critically evaluate these discoveries, highlight their immunological implications, and discuss the translational potential of targeting this axis for AD diagnosis and therapy.

## 2 miR-32533: a novel multifunctional therapeutic target for AD

The study by our group provides robust evidence that establishes miR-32533 as a central regulator in AD (16–19). Its identification by RNA sequencing in amyloid precursor protein (APP)/Presenilin-1 dE9 (PS1) mouse brains and rigorously validated as a canonical Drosha/Dicer product via northern blot, fluorescence *in situ* hybridization (FISH), and quantitative real-time polymerase chain reaction (qRT-PCR). Several critical characteristics make it a promising therapeutic candidate:

### 2.1 miR-32533 is brain-enriched and early dysregulated in AD

The expression of miR-32533, enriched in the cortex and hippocampus, is significantly reduced in AD models, exemplified by APP/PS1 and 5×familial AD (5×FAD) mice, and APPswe cells (16). This downregulation is also observed in the plasma of AD patients (16). The nadir of its expression at 5 months in mice suggests it may be an early event in the disease progression, making it a potential biomarker for early detection.

### 2.2 miR-32533 levels correlate with clinical parameters

The positive correlation between plasma miR-32533 levels and minimum mental state examination (MMSE) scores in AD patients, along with its significant diagnostic value (Area Under Curve (AUC)=0.737), featuring 53.8% sensitivity and 91.7% specificity, strengthens its clinical importance. Intriguingly, its association with the Aβ_1-42_/Aβ_1–40_ ratio, but not with tau phosphorylation at threonine 217 (p-Tau217), places it within the amyloid pathology cascade (16).

### 2.3 miR-32533 overexpression exhibits pleiotropic neuroprotective effects

Functionally, overexpression of miR-32533 has demonstrated a remarkable capacity to alleviate Aβ-induced neurotoxicity both *in vitro* and *in vivo*. It improves neuronal viability, reduces oxidative stress by decreasing ROS and malondialdehyde (MDA) while increasing glutathione (GSH) and superoxide dismutase (SOD), and inhibits mitochondrial apoptosis (16). Mechanically, miR-32533 overexpression elevates the ratio of anti-apoptotic markers, reduces cytochrome *c* expression, and decreases the cleaved cysteinyl aspartate-specific proteinase-3 (caspase-3) to caspase-3 ratio (16). Notably, miR-32533 overexpression also significantly suppresses the release of key pro-inflammatory cytokines TNF-α and IL-6, demonstrating its immunomodulatory capacity (16). Conversely, all these protective effects are reversed upon suppression of miR-32533 expression (16). Given the established role of microRNAs in synaptic plasticity (20), miR-32533 is a putative regulator of this process. The multi-faceted nature of the protective effects of miR-32533 is exactly what is desirable for a complex disease like AD.

## 3 The miR-32533/CREB5 axis: a novel regulator of AD pathogenesis and neuroinflammation

**Figure 1:**
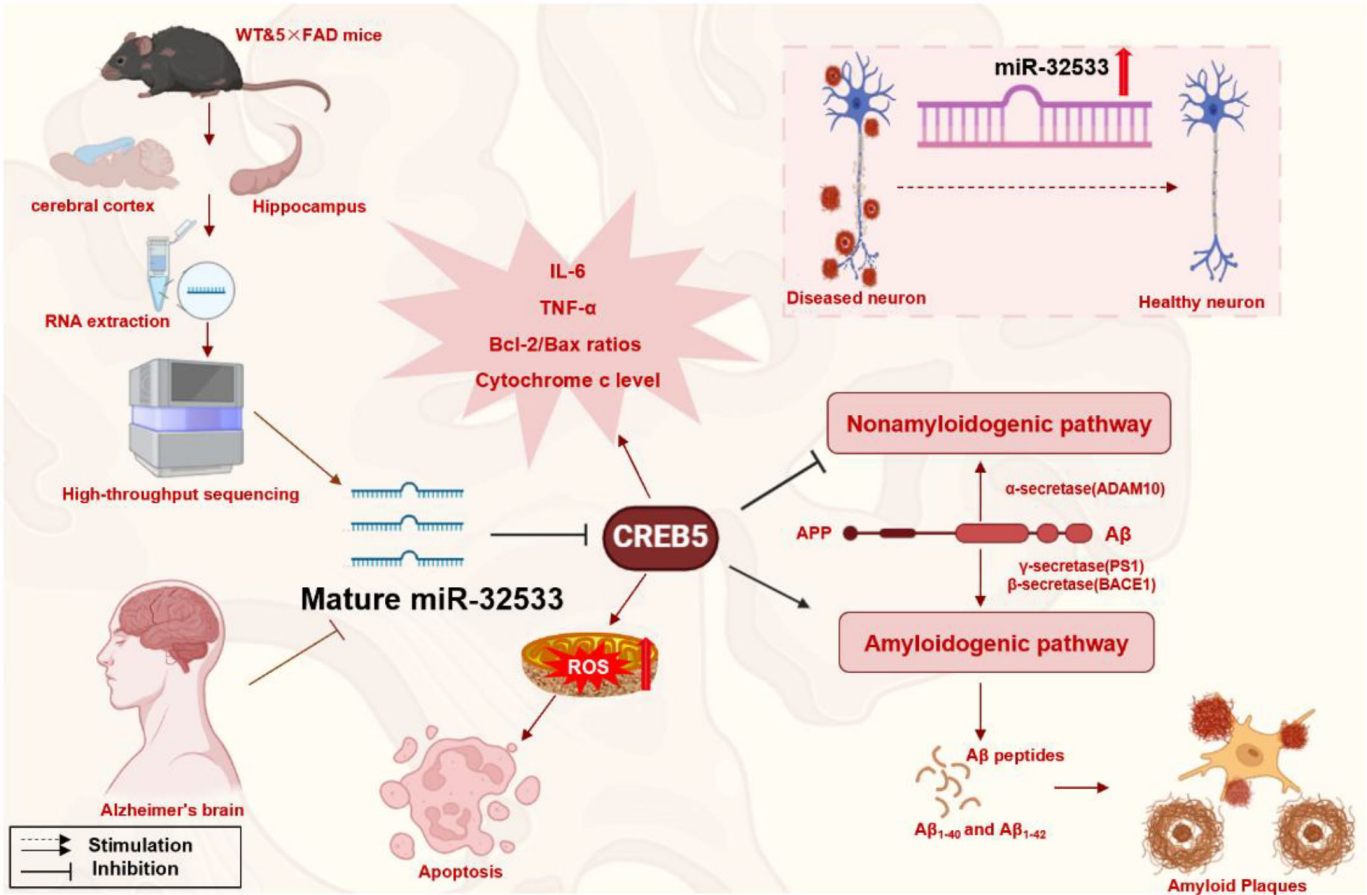
Role of miR-32533/CREB5 in AD pathology. AD, alzheimer’s disease; CREB5, cyclic-AMP response binding protein 5; Aβ, amyloid-beta; ADAM10, a disintegrin and metalloproteinase 10; PS1, presenilin-1; APP, amyloid precursor protein; BACE1, beta-site cleaving enzyme 1; IL-6, interleukin-6; TNF-α, tumor necrosis factor-alpha. (Created using BioRender.com).

The most significant mechanistic insight from our study is the elucidation of CREB5 as a direct and functional target of miR-32533 (16). This axis represents a master regulatory switch for Aβ metabolism and its downstream consequences (**Figure 1**).

### 3.1 CREB5 is directly targeted for transcriptional regulation

Our research reveals that miR-32533 binds to the 3’ untranslated region (3’-UTR) region of CREB5 mRNA, thereby repressing its expression. Our multi-omics approach revealed CREB5 as the only predicted miR-32533 target gene showing significant upregulation in two distinct AD mouse models (5×FAD, *p* = 0.022; APP/PS1, *p* = 0.038) (16). Our results are reinforced by Tian et al., who identified CREB5 as a factor critically associated with neurodegenerative disease and aging (21). CREB5 acts as a transcriptional hub, shifting amyloid processing by directly repressing a disintegrin and metalloproteinase 10 (ADAM10) and activating beta-site cleaving enzyme 1 (BACE1)/PS1 through promoter binding (16). This dual action elegantly explains how miR-32533 downregulation leads to Aβ overproduction.

### 3.2 The miR-32533/CREB5 axis is specifically associated with amyloid pathology

Aβ deposition, as the initiating factor in AD, promotes aging-associated DNA damage (22). The association of CREB5 with brain atrophy and Aβ accumulation reinforces our findings (23). Notably, in the tested AD models, the miR-32533/CREB5 axis exerted a significant effect on Aβ-related pathologies but had no remarkable effect on tau phosphorylation. Our findings indicate that the miR-32533/CREB5 axis attenuates Aβ_1–42_ generation through the coordinated regulation of ADAM10 and BACE1/PS1. Cognitive deficits and elevated Aβ levels in AD models were linked to the dysregulation of this axis, whereas tau pathology remained unaffected (16). This specificity suggests a primary role in the amyloid cascade, potentially rendering it most relevant for early-stage AD or as a combination therapy with tau-targeting agents in the later disease stages.

### 3.3 The miR-32533/CREB5 axis is a bridge of amyloid pathology and neuroinflammation

Beyond Aβ regulation, the miR-32533/CREB5 axis is critically involved in neuroinflammation. Our study shows that modulation of this axis directly affects the levels of pro-inflammatory cytokines IL-6 and TNF-α. Although initially studied in oncology (24, 25), CREB5 is increasingly implicated in neurodegenerative diseases, including AD, where its expression is significantly elevated (16, 26, 27). As a disease node, suppressing CREB5 could inhibit tumorigenesis (24), while upregulation of CREB5 counteracts the anti-apoptotic activity in Parkinson’s disease models (28). The involvement of CREB5 in apoptosis and inflammation is exemplified in sepsis-induced acute kidney injury, where the CREB5/NF-κB axis serves as a crucial downstream effector of FOXQ1 (29). Given that CREB5 has been involved in the TNF signaling pathway in other contexts (29, 30), its upregulation in AD likely exacerbates the chronic neuroinflammatory response.

By repressing CREB5, miR-32533 can concurrently reduce Aβ production and mitigate neuroinflammation, disrupting the detrimental cycle between Aβ accumulation and glial activation. Inhibition of miR-32533 leads to CREB5 upregulation, leading to ROS-driven oxidative stress, apoptosis (decreased B cell lymphoma 2 (Bcl-2)/Bcl2-association X protein (Bax) ratio and increased cytochrome *c* expression), and elevates pro-inflammatory cytokines (IL-6 and TNF-α) (16). CREB5 overexpression reverses these protective effects, increasing ROS and MDA, decreasing GSH and SOD activity, and increasing neuroinflammation (16). Supporting this, miR-769-5p protects against AD by targeting CREB5 to alleviate oxidative stress (31), and CREB5 modulates neuroinflammation and cognition in Human Immunodeficiency Virus (HIV) encephalitis (32). Collectively, the miR-32533/CREB5 axis connects amyloidosis and immunopathology in AD.

## 4 Targeting miR-32533: a strategic approach for AD therapy

Accumulating preclinical studies indicate that dysregulated miRNA expression in AD patients, especially in plasma, brain tissues, and synaptic regions, contributes to cognitive impairments (33–35). Regarding discoveries of the miR-32533/CREB5 axis, two primary therapeutic strategies can be employed: restoring miR-32533 function or inhibiting CREB5 activity.

The utilization of advanced, brain-penetrant delivery systems, such as optimized adeno-associated virus (AAV) vectors, exosomes and nanoparticles (36–42), to administer miR-32533 mimics constitutes a direct gene therapy approach. *In vivo* studies using AAV-loaded mimics in APP/PS1 mice have shown promising results, demonstrating restored cognitive function, reduced Aβ burden, and mitigated neuroinflammation (16–19). The primary challenge lies in ensuring efficient, safe, and long-lasting delivery to the central nervous system (CNS).

An alternative strategy involves developing small-molecule inhibitors capable of disrupting the transcriptional activity of CREB5, especially its binding to the BACE1 and PS1 promoters. This approach could replicate the benefits of miR-32533 upregulation, while avoiding the delivery difficulties associated with oligonucleotides. High-throughput screening and structure-based drug design could be utilized to identify these compounds (43–48).

Given the immunomodulatory effects of this axis, a combined treatment using a miR-32533-based agent alongside passive anti-Aβ immunotherapy might exhibit synergistic effects. While immunotherapy eliminates existing plaques, restoring miR-32533 could reduce the formation of new Aβ plaques and, at the same time, mitigate the associated neuroinflammatory response, potentially leading to enhanced clinical effectiveness.

## 5 Discussion and future perspectives

Our research group identified miR-32533, a 23-nucleotide miRNA with negligible coding capacity, presenting a promising diagnostic candidate. The assessment of its aberrant expression using receiver operating characteristic (ROC) curves shows significant predictive value for AD, supporting its potential as a non-invasive biomarker. Furthermore, our findings reveal a tight regulatory relationship between miR-32533 and its direct target, CREB5, indicating that the miR-32533/CREB5 axis is a critical regulator of Aβ metabolism, oxidative stress, and neuroinflammatory responses. To strengthen our findings, subsequent studies will employ the physiologically relevant brain organoid model (49). Moreover, to apply miR-32533/CREB5 axis clinically, several questions remain to be addressed.

First, it is essential to conduct large-scale longitudinal studies on human cohorts to confirm the diagnostic and prognostic significance of plasma miR-32533. Given that miR-32533 likely targets other molecules besides CREB5, a comprehensive identification of its entire target network using techniques such as Argonaute CrossLinking ImmunoPrecipitation followed by sequencing (AGO-CLIP-seq) will provide systems-level insight into its function and uncover potential off-target effects and additional synergistic pathways (50–52).

Second, although the present study did not identify a connection to tau, it is of utmost importance to explore this aspect in alternative models, particularly those with robust tauopathy. The interaction among Aβ, inflammation, and tau is central to AD, and understanding whether and how this axis impacts tau pathogenesis in the later stages of the disease is crucial (53–55).

Third, the primary obstacle to any CNS-targeting miRNA therapy is its delivery (56–58). It is paramount to invest in the development of novel, cell-type-specific delivery platforms. Moreover, comprehensive toxicology studies are necessary to evaluate the long-term safety of chronically modulating this axis, considering the potential roles of CREB5 in other tissues and processes.

## 6 Final considerations

In conclusion, the identification of the miR-32533/CREB5 pathway unveils a previously unknown regulatory mechanism in AD pathogenesis, uniquely linking Aβ metabolism with neuroinflammation. Its capability to coordinately modulate multiple aspects of AD pathology, ranging from secretase balance to oxidative stress and cytokine release, renders it an exceptionally promising therapeutic target. Looking ahead, it will be imperative to make a concerted effort to integrate rigorous basic scientific research with thorough mapping of the miR-32533 regulatory network, alongside the development of innovative bioengineering delivery methodologies. By focusing on this endogenous regulatory pathway, we might develop the next wave of multi-target, disease-modifying therapies for AD, which not only clear pathological proteins but also alleviate the destructive inflammatory environment in the brain.
